# Right Atrial Pressure Affects the Interaction between Lung Mechanics and Right Ventricular Function in Spontaneously Breathing COPD Patients

**DOI:** 10.1371/journal.pone.0030208

**Published:** 2012-01-17

**Authors:** Bart Boerrigter, Pia Trip, Harm Jan Bogaard, Herman Groepenhoff, Frank Oosterveer, Nico Westerhof, Anton Vonk Noordegraaf

**Affiliations:** 1 Department of Pulmonary Diseases, VU University Medical Center, Amsterdam, The Netherlands; 2 Department of Physiology, Institute for Cardiovascular Research, VU University Medical Center, Amsterdam, The Netherlands; Temple University, United States of America

## Abstract

**Introduction:**

It is generally known that positive pressure ventilation is associated with impaired venous return and decreased right ventricular output, in particular in patients with a low right atrial pressure and relative hypovolaemia. Altered lung mechanics have been suggested to impair right ventricular output in COPD, but this relation has never been firmly established in spontaneously breathing patients at rest or during exercise, nor has it been determined whether these cardiopulmonary interactions are influenced by right atrial pressure.

**Methods:**

Twenty-one patients with COPD underwent simultaneous measurements of intrathoracic, right atrial and pulmonary artery pressures during spontaneous breathing at rest and during exercise. Intrathoracic pressure and right atrial pressure were used to calculate right atrial filling pressure. Dynamic changes in pulmonary artery pulse pressure during expiration were examined to evaluate changes in right ventricular output.

**Results:**

Pulmonary artery pulse pressure decreased up to 40% during expiration reflecting a decrease in stroke volume. The decline in pulse pressure was most prominent in patients with a low right atrial filling pressure. During exercise, a similar decline in pulmonary artery pressure was observed. This could be explained by similar increases in intrathoracic pressure and right atrial pressure during exercise, resulting in an unchanged right atrial filling pressure.

**Conclusions:**

We show that in spontaneously breathing COPD patients the pulmonary artery pulse pressure decreases during expiration and that the magnitude of the decline in pulmonary artery pulse pressure is not just a function of intrathoracic pressure, but also depends on right atrial pressure.

## Introduction

An imposed high intrathoracic pressure can depress venous return and subsequently right ventricular output [1], [2], particularly in patients with a low fluid status [3], [4]. In chronic obstructive pulmonary disease (COPD), airway obstruction leads to an increase in intrathoracic pressures during expiration due to the use of expiratory muscles and lung hyperinflation. It is unknown whether similar interactions between intrathoracic pressure, right heart filling, right heart output and fluid status exist in spontaneously breathing COPD-patients. Many patients with COPD have evidence of fluid retention and an increased right atrial pressure (RAP), and diuretics are frequently prescribed to restore fluid balance. It may be argued that spontaneously breathing COPD patients with a low right atrial pressure are more vulnerable for an impaired right heart filling during expiration, possibly translating into an impaired RV output [5].

In order to better understand cardiopulmonary interactions in COPD, one would ideally measure beat-to-beat changes in right ventricular stroke volume over the respiratory cycle and relate changes in intrathoracic pressures to changes in stroke volume. Because such measurements are technically difficult, we used the decline in pulmonary artery pulse pressure to describe the effect of expiratory airflow limitation on RV return and output in spontaneously breathing COPD patients at rest and during exercise. We show that in COPD, like in patients on positive pressure ventilation, the interplay between intrathoracic pressure and right atrial pressure critically determines RV output.

## Methods

### Patients

All patients participated in a study to investigate the effects of dynamic hyperinflation on the pulmonary circulation and right ventricular function and therefore underwent right heart catheterization and oesophageal pressure measurement at rest and during exercise. Eligible patients were diagnosed with moderate to very severe COPD, defined as an post-bronchodilator forced expiratory volume in one second (FEV_1_)/forced vital capacity (FVC) ≤0.7 and a FEV_1_-value < 80% or predicted, according to ATS/ETS criteria [6] Exclusion criteria were a history of left sided cardiac failure, signs of left ventricular dysfunction and/or valvular disease on Doppler echocardiography, neuromuscular disorders or an acute exacerbation in the 4 weeks prior to inclusion.

This study has institutional review board approval (Medical ethical review committee VU University Medical Center, registration number: NL30766.029.10) and all patients were informed and gave written consent to the procedures.

*For comparison we used data from patients with normal pulmonary function and normal pulmonary hemodynamics, analysed in our center for suspected pulmonary hypertension.*

### Right heart catheterization

A balloon-tipped, flow directed 7.5 Fr, triple-lumen Swan-Ganz catheter was brought, via the jugular vein, into position under local anaesthesia. The patients were in stable condition, lying supine and breathing room air, while heart rate was continuously monitored. The ports of the catheter were positioned in the right atrium, right ventricle and pulmonary artery. An arterial line was inserted in the radial artery.

### Oesophageal pressure

We continuously monitored oesophageal pressure, as a measure of intrathoracic pressure. A standard oesophageal balloon-catheter (Microtek Medical B.V. Zutphen, The Netherlands) was inserted transnasally with the use of lidocaine gel (2%) and positioned in the mid-oesophagus. Subsequently, 5 mL of air was injected into the balloon followed by the withdrawal of 4 mL to partially inflate the balloon. The pressure signal was checked and in case of cardiac beat artefacts the position of the balloon-catheter was slightly adjusted upwards. Pleural pressure measurements were not available in the group of control subjects, but careful inspection of the pulmonary artery and right atrial pressure curves allowed identification of in- and expiratory phases.

### Protocol

With the Swan-Ganz catheter, oesophageal balloon-catheter and arterial line in position patients were placed on an electromagnetically braked cycle ergometer (Ergoline GmbH, Bitz, Germany), in supine position. The exercise protocol consisted of 3 minutes rest, followed by a progressively increasing work rate to maximum tolerance. Work rate was increased every 3 minutes until maximum. The different levels of exercise were based on a maximal exercise test performed the day before.

### Measurements

Pressures in the right atrium, right ventricle, pulmonary artery, oesophagus and radial artery were simultaneously measured and digitally recorded using a PowerLab data acquisition system (ADInstruments). Oxygen consumption (VO_2_) was measured during the entire protocol using a metabolic cart (Vmax 229, Viasys, Yorba Linda, CA, USA). At rest and peak exercise, measurements of pulmonary capillary wedge pressure (PCWP) and inspiratory capacity (IC) were performed and blood samples were collected. Afterwards, cardiac output (CO) obtained by direct Fick-method from arterial- and mixed venous oxygen saturation and VO_2_. Pulmonary vascular resistance (PVR) was calculated as (mPAP - PCWP)/CO. Mean right atrial pressure and oesophageal pressure, both at end-expiration, were used for calculating right atrial transmural pressure (RAP_tm).

A semiautomatic program using Matlab R2008a (The MathWorks, Natick, MA) was used to acquire systolic and diastolic pressure in the pulmonary artery beat by beat over at least 10 consecutive respiratory cycles of the resting period and during the last minute of exercise. The decline in pulse pressure in the pulmonary artery was calculated as the pulse pressure of the last heartbeat during expiration (*pulse 2*) minus the pulse pressure of the first heartbeat during expiration (*pulse 1*) and represented as percentage of *pulse 1*. Mean pulmonary artery pressure was calculated on a beat-to-beat basis. The change in systolic pressure in the radial artery over the respiratory cycle was calculated as the lowest minus the highest systolic pressure.

### Lung function

Lung function measurements were performed within two days of the right heart catheterization. Static and dynamic lung volumes and diffusion capacity of carbon monoxide (Dlco) using a metabolic chart (Vmax 229, Viasys, Yorba Linda, CA, USA) were measured according to the European Respiratory Society guidelines [7], [8].

### Statistical analysis

Lung function, exercise and hemodynamic values are presented as mean ± standard deviations. Comparison of rest and exercise was performed with a paired sample t-test. To evaluate the influence of RAP_tm on the decline in pulse pressure in the pulmonary artery a non-linear curve was fitted through the data points and correlation coefficients (r^2^) were calculated. All tests were two-tailed and a P-value of <0.05 was considered significant. Analyses were performed with SPSS 15.0 for Windows (SPSS Inc, Chicago Illinois) or GraphPad-Prism for Windows (version 4.0).

## Results

### Patient characteristics

Twenty-one patients (13 female) with a mean age of 63 years were included in this study. Pulmonary function characteristics are shown in table 1. In the study group FEV_1_ ranged from 0.58 to 2.72 L. and the FEV_1_/VC ratio ranged from 27 to 64%. Based on the FEV_1_ percent of predicted, 9 patients had moderate, 10 had severe and 2 had very severe airway obstruction. The resting and peak exercise metabolic parameters are shown in table 2. Maximal power ranged from 10 to 120 Watt, with a mean of 38 Watt. Peak VO2 and maximal ventilation (VEmax) were low compared to the predicted values. The hemodynamic measurements at rest and during peak exercise are summarized in table 3. Thirteen of the 21 patients were diagnosed with pulmonary hypertension (resting mean pulmonary artery pressure >25 mmHg [9]. Cardiac index ranged from 1.4 to 4.7 L/min/m^2^. During exercise pulmonary artery pressures increased significantly. The demographic, pulmonary function and hemodynamic characteristics of the control subjects are summarized in table 4.

**Table 1:** Lung function characteristics.

Variable	Mean ± SD	% of predicted ± SD
**FEV_1_, L**	1.49±0.58	53±16
**FEV_1_/VC, %**	45±11	
**VC, L**	3.33±1.0	96±20
**TLC, L**	6.53±1.12	110±17
**RV/TLC, %**	49±12	
**FRC, L**	4.33±1.0	141±35
**DLCO, mmol/kPA/min**	3.86±1.53	44±15
**PaO_2_, mmHg**	64±14	
**PaCO_2_, mmHg**	40±8	

FEV_1_: Forced Expiratory Volume in 1^st^ second, VC: Vital Capacity, TLC: Total Lung Capacity, RV: Residual Volume, FRC: Functional Residual Capacity, DLCO: Diffusion Capacity of the Lungs for Carbon Monoxide.

**Table 2:** Exercise test parameters.

Variable	Rest	Peak Exercise
VO_2_, mL/min	286±65	841±327*
VO_2_, ml/kg/min	3.8±0.6	11±3.45*
VE, L/min	13±3.4	35±16*
VE/MVV, %	42±19	59.4±16*
Vt, L	0.73±0.25	1.14±0.56*
RR, breaths/min	18±4	32±8*
IC, L	2.20±1.57	1.57±0.55*

VO_2_: Oxygen uptake, VE: Minute ventilation, MVV: Maximal Voluntary Ventilation, Vt: Tidal Volume, RR: Respiratory Rate, IC: Inspiratory Capacity,

*p<0.05 versus rest.

**Table 3:** Hemodynamic parameters.

Variable	Rest	Peak Exercise
mPAP, mmHg	32±14	57±16*
CI, L/m^2^	3.3±0.9	5.5±1.5*
HR, b/min	83±16	120±18*
SVI, ml	40±11	46±12*
PVR	348±340	365±308
mRAP, mmHg	7±5	15±6*
PCWP, mmHg	9±2	16±5*
SaO_2_, %	91±5	84±9*
SvO_2_, %	63±10	39±8*

mPAP: mean Pulmonary Artery Pressure, CI: Cardiac Index, HR: Heart Rate, SVI: Stroke Volume Index, PVR: Pulmonary Vascular Resistance, mRAP: mean Right Atrial Pressure, PCWP: Pulmonary Capillary Wedge Pressure, SaO_2_: Arterial oxygen saturation, SvO_2_: Mixed venous oxygen saturation.

*p<0.05 versus rest.

**Table 4:** Characteristics of control subjects.

Variable	
Male/Female	3/7
Age, yr	48±10
Height, cm	169±9
Weight, kg	76±14
FEV_1_, L	3.09±1.12
FEV_1_, % of predicted	108±19
FEV_1_/VC, %	77±5
VC, % of predicted	112±30
DLCO, % of predicted	95±17
mPAP, mm Hg	17±4
CI, Lmin/m^2^	4.1±0.8
PVR, dyn/s/cm^−5^	88±38

FEV_1_: Forced Expiratory Volume in 1st second, VC: Vital Capacity, DLCO: Diffusion Capacity of the Lungs for Carbon Monoxide, mPAP: mean Pulmonary Artery Pressure, CI: Cardiac Index, PVR: Pulmonary Vascular Resistance.

### Oesophageal and transmural right atrial pressure

End expiratory oesophageal pressure was 4±4 mmHg at rest and increased to 12±4 mmHg at peak exercise, showing expiratory airflow obstruction. End inspiratory pressure was −6±3 mmHg at rest and decreased to −13±3 at peak exercise. Mean right atrial pressure was 8±4 mmHg and increased significantly with exercise to 17±8 mmHg.

**Figure 1:**
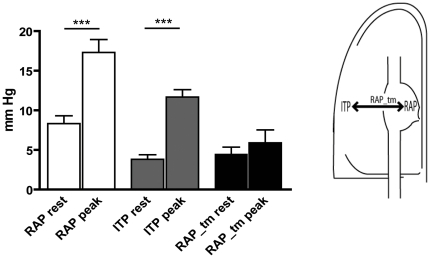
The right atrial (RAP) and intrathoracic pressure (ITP) at rest and peak exercise. Note the rise in RAP and ITP as the transmural pressure of the right atrium (RAP_tm) remains constant. RAP_tm is calculated as pressure inside the right atrium (RAP) minus the pressure outside the right atrium, which is the ITP. *** = P<0.0001.

Transmural right atrial pressure (RAP_tm) during expiration ranged from 1 to 16 mmHg (mean 6) at rest. During exercise, oesophageal pressure and right atrial pressure increased with a similar amount and thus, RAP_tm during exercise was similar to RAP_tm measured at rest (see also figure 1).

### Expiratory pulse pressure decline in the pulmonary artery

**Figure 2:**
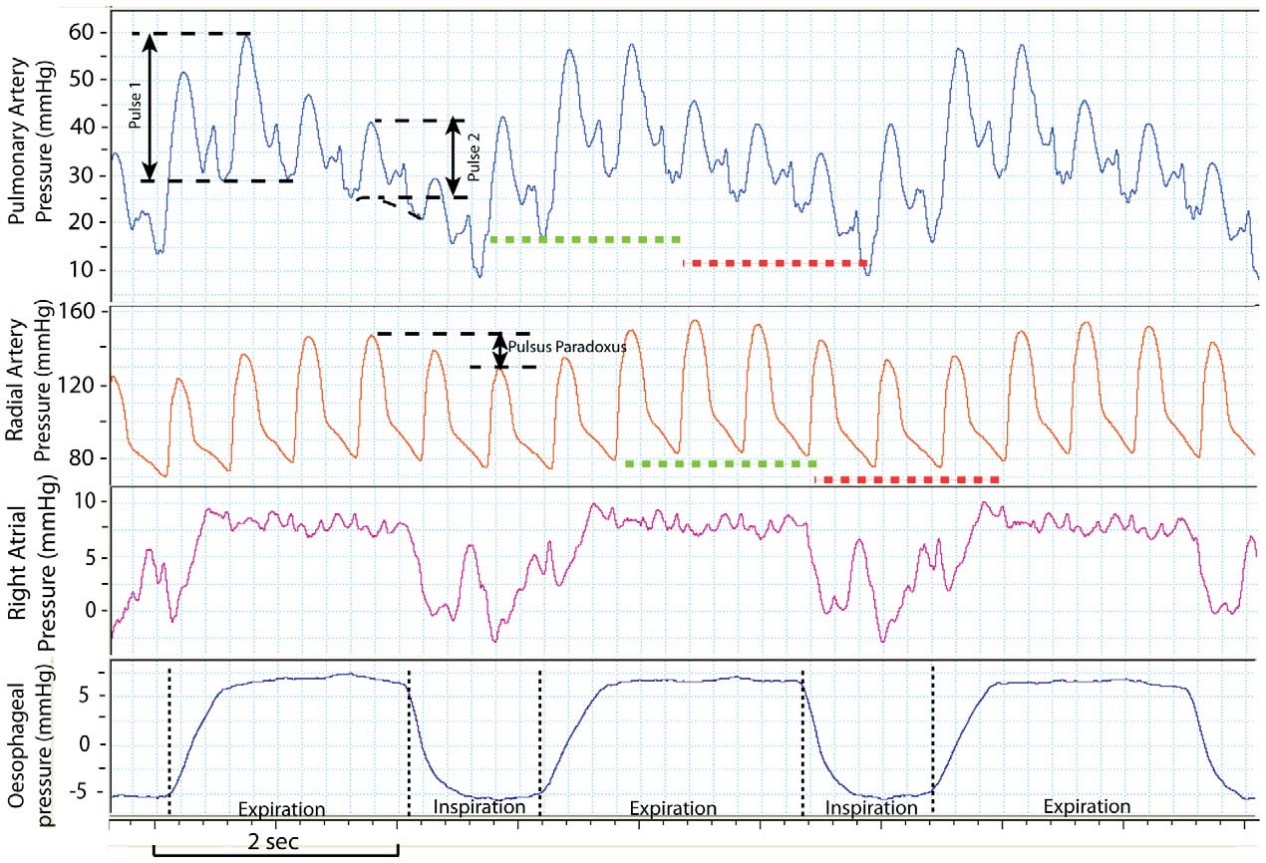
Example of the behavior of the pulmonary artery pressure over the respiratory cycle in a COPD patient. In the upper channel we show the decline in pulse pressure (pulse 1 - pulse 2) in the pulmonary artery during expiration, which is a consistent phenomenon over all respiratory cycles. The second channel shows the pressure in the radial artery. The decline in pulse pressure in the radial artery seems to follow the decline in the pulmonary artery pressure. (red dotted lines). During expiration, the intrathoracic pressure (channel 4) probably exceeds the central venous pressure, which would explain the flat line of the right atrial pressure (channel 3).

**Figure 3:**
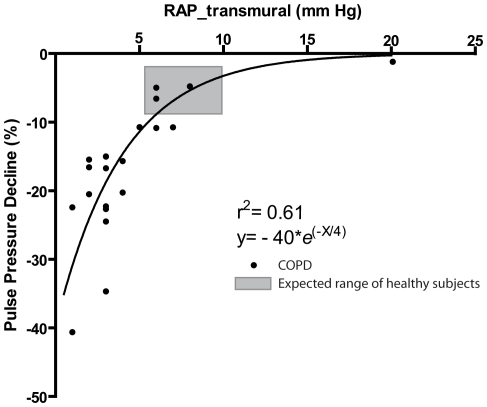
Relationship between transmural pressure of the right atrium (RAP_tm) and the percent decline in pulse pressure in the pulmonary artery. Gray area represents the suspected window of normal, based on the calculated pulse pressure decline and the suspected RAP_tm of the control subjects.

During expiration there was a decline in the pulmonary artery pulse pressure in all but one patient. The patient not showing a decline in pulmonary artery pulse pressure had severe pulmonary hypertension. The decline in pulse pressure as shown in figure 2 ranged from 0 to 41% with a mean of 17% and was greatest in those patients with a low RAP_tm (see figure 3). The relationship between pulse pressure decline and RAP_tm could be described by a natural logarithmic curve (best-fit correlation; r^2^ = 0.61). The decline in pulse pressure in the pulmonary artery during *expiration* was followed by a decrease in systolic radial artery pressure during *inspiration*, a phenomenon which is also known as pulsus paradoxus (see figure 2). The average expiratory decline in pulse pressure in the healthy subjects was −4.5%±3.1%, which was significantly lower than the pulse pressure decline in COPD-patients (unpaired t-test, p: 0.0012).

### Expiratory pulse pressure decline during exercise

We subsequently examined the presence of similar relationships between intrathoracic, right atrial and pulmonary artery pulse pressures during exercise. As shown in figure 1, both right atrial pressure and intrathoracic pressure increased significantly during exercise, which resulted in an unaltered RAP_tm. With exercise, there was an expiratory decline in mean pulmonary artery pressure and pulmonary artery pulse pressure, ranging from 0 to 35% with a mean of 20%. Again, there was an exponential correlation between the expiratory pulmonary artery pulse pressure decline and RAP_tm (r^2^ = 0.52).

## Discussion

This is the first study showing a significant interaction between intrathoracic pressure, right atrial filling and right ventricular output in spontaneously breathing COPD patients. Based on combined measurements of intrathoracic and hemodynamic pressures in 21 patients with moderate to very severe airflow obstruction, we show that the pulmonary artery pulse pressure decreases during expiration and that the magnitude of this decline is not just a function of intrathoracic pressure, but also depends on right atrial transmural pressure. The interaction between intrathoracic pressure, right atrial filling and right ventricular output is well known in mechanically ventilated patients: when high levels of positive airway pressure are required to maintain oxygenation, venous return may be impaired [3], [4] and fluid administration may be necessary to prevent hemodynamic collapse.

Intrathoracic pressure is increased during expiration in patients with COPD, which leads to an impairment of caval blood entering the thorax [10], [11]. Here we show the hemodynamic consequence, which is an expiratory drop in mean pulmonary artery pressure and pulmonary artery pulse pressure. Because the intrathoracic pressure remained unaltered and pulmonary arterial compliance can be considered constant during expiration [12], the drop in pulmonary artery pressure implies a decreased right ventricular stroke volume during expiration in COPD. While under physiological circumstances the RV and pulmonary circulation are capable of buffering most respiratory variations in venous return towards a near constant flow [13], [14], [15], the impairment of venous return during expiration seem to be exaggerated to such a degree that a drop in RV stroke volume and pulmonary artery pressure is created due to expiratory airflow limitation.

**Figure 4:**
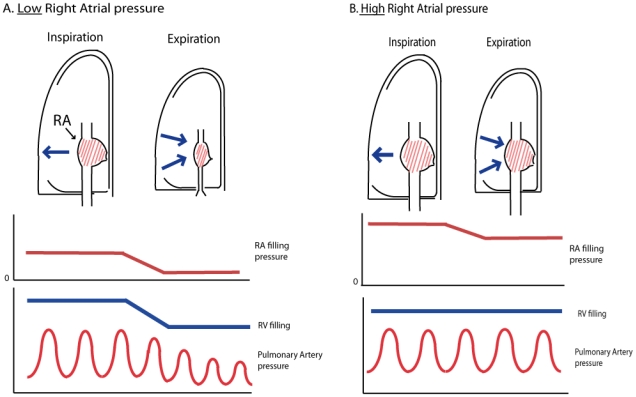
Schematic illustration of the effect of similar intrathoracic pressures (blue arrows) on right atrial (RA) filling, RV filling and the pulmonary artery pressure in a situation of either a low (A) or high (B) right atrial pressure. When RAP is low, the positive intrathoracic pressure during expiration leads to an impaired venous return and a variation in RV filling and stroke volume during the respiratory cycle. When RAP pressure is high, the right atrium acts as a reservoir which maintains RV filling and ensures a stable pulmonary artery pulse pressure and RV stroke volume during expiration.

The decline of the pulmonary artery pulse pressure was largest in patients with a normal right atrial pressure (fig. 3). A normal right atrial pressure in combination with a high intrathoracic pressure means a low transmural pressure of the right atrium. The pulsatility of the right atrial pressure during expiration decreased in patients with a normal right atrial pressure (fig. 2), indicating a serious impairment of right atrial filling. In these patients the right atrial pressure equals the rise in intrathoracic pressure which is the major cause of a decline in venous return [16], [17]. In contrast, the expiratory decline in pulmonary artery pulse pressure was attenuated or completely absent in COPD patients with a high right atrial pressure. As illustrated in figure 3 and exemplified in figure 4, a high pressured and dilated right atrium and caval vein can act as a reservoir maintaining constant RV filling during expiration despite any increases in intrathoracic pressure. In our study group the highest right atrial pressures were found in patients with pulmonary hypertension. In COPD patients with associated pulmonary hypertension, both RV afterload and preload are increased [18], [19], [20], [21]. Paradoxically, in patients with COPD associated pulmonary hypertension, the impaired RV function seems to prevent a decline in RV output during expiration via distension of the right atrium. We found a natural logarithmic relationship between RAP_tm and the expiratory pulse pressure, which can be explained by the inability of the RAP_tm to reach negative values and the inability of the pulmonary artery pulse pressure change during expiration to reach positive values.

The average expiratory decline in pulse pressure in the 10 healthy subjects was −4.5%. Right atrial pressure in these subjects was 5±2 mm Hg. When we assume a pleural pressure at the end of expiration of −2 to −4 mmHg (as has been previously shown in healthy subjects [22]), the RAP_tm was between 7 and 9 mm Hg. These values fit nicely on the line drawn in figure 3. This range in RAP_tm may represent a window allowing optimal RV filling. It can be hypothesized that some degree of fluid retention in COPD is an adequate response to maintain RAP_tm close to the physiological window of 7 to 9 mmHg. Alternatively, it can be hypothesized that the higher RAP is just the consequence of an increased RV afterload.

We found a comparable interaction between intrathoracic and intravascular pressures during exercise, which is likely explained by the fact that the same hemodynamic mechanisms are at work at rest and during exercise (figure 1). The increase in RAP during exercise is likely the result of an increased intrathoracic pressure, leading to an unaltered filling pressure of right atrium. It has to be noted, however, that the patients in our study performed supine exercise and it is possible that during upright exercise (such as during normal daily activities), venous return may be lower. In this situation, the negative effect of a high intrathoracic pressure during expiration may be larger.

We observed a drop in systemic blood pressure over the respiratory cycle in all patients with an expiratory drop in pulmonary artery pulse pressure. The drop in pulse pressure in the systemic circulation followed about 2–3 heartbeats after the decline in the pulmonary artery pulse pressure and, therefore, occurred partially during inspiration (see fig. 2). We did not study whether the changes in systemic pressure were causally related to the decline in the pulmonary artery pulse pressure, but it can be speculated that in patients with airflow obstruction part of the phenomenon of a pulsus paradoxus (>10 mmHg drop in systemic pressure during *inspiration*), is due to an interaction between right and left ventricular performance. Recently, even mild COPD patients were shown to have a reduced left ventricular end diastolic volume and stroke volume [23]. In this study from Barr et al. on a very large cohort of normal subjects and patients with mild COPD, left ventricular dimensions were correlated to FEV_1_. We speculate that this observation may be partially explained by a sequence starting from airway obstruction leading to increased intrathoracic pressure, subsequent underfilling of the right ventricle and finally, underfilling of the left ventricle and a decrease in stroke volume. The possible role of fluid status and the use of diuretics on the observed relationship between FEV_1_ and cardiac dimensions in the patients studied by Barr et al. was addressed in the correspondence which followed the original publication [5].

It is conceivable that the hemodynamic impairment in COPD can be described as a continuum between two extremes. On the one hand, there are patients with a low pulmonary artery pressure in whom a normal right atrial pressure translates into impaired right atrial filling and a low right ventricular output at the end of expiration. On the other hand, there are COPD patients with an elevated pulmonary artery pressure, who are functionally impaired by a high afterload but have a normal right ventricular preload. In these patients, stroke volume is impaired during the entire respiratory cycle, as the elevated right atrial pressure maintains a constant filling pressure of the right ventricle. The heterogeneity of the disease implies that individual COPD patients may have different combinations of preload and afterload impairments.

In conclusion, we here show that in COPD, the pulmonary artery pulse pressure declines during expiration, and that this effect is most prominent in patients with a low right atrial filling pressure. This implies that expiratory airflow limitation leads to expiratory blood flow limitation in the pulmonary circulation. Impaired right atrial filling during expiration may explain part of the impairment in resting and exercise stroke volume in COPD patients.
